# A hydrophobic proline-rich motif is involved in the intracellular targeting of temperature-induced lipocalin

**DOI:** 10.1007/s11103-015-0326-x

**Published:** 2015-05-10

**Authors:** Francesc Hernández-Gras, Albert Boronat

**Affiliations:** Departament de Bioquímica i Biologia Molecular, Facultat de Biologia, Universitat de Barcelona, Avda. Diagonal 643, 08028 Barcelona, Spain; Centre de Recerca en Agrigenòmica (CRAG), Consorci CSIC-IRTA-UAB-UB, Campus Universitat Autònoma de Barcelona, Bellaterra-Cerdanyola del Vallès, 08193 Barcelona, Spain

**Keywords:** Lipocalin, Temperature-induced lipocalin, Protein targeting, Membrane, Arabidopsis, Tomato

## Abstract

Temperature-induced lipocalins (TILs) play an essential role in the response of plants to different abiotic stresses. In agreement with their proposed role in protecting membrane lipids, TILs have been reported to be associated to cell membranes. However, TILs show an overall hydrophilic character and do not contain any signal for membrane targeting nor hydrophobic sequences that could represent transmembrane domains. Arabidopsis TIL (AtTIL) is considered the ortholog of human ApoD, a protein known to associate to membranes through a short hydrophobic loop protruding from strands 5 and 6 of the lipocalin β-barrel. An equivalent loop (referred to as HPR motif) is also present between β-strands 5 and 6 of TILs. The HPR motif, which is highly conserved among TIL proteins, extends over as short stretch of eight amino acids and contains four invariant proline residues. Subcellular localization studies have shown that TILs are targeted to a variety of cell membranes and organelles. We have also found that the HPR motif is necessary and sufficient for the intracellular targeting of TILs. Modeling studies suggest that the HPR motif may directly anchor TILs to cell membranes, favoring in this way further contact with the polar group of membrane lipids. However, some particular features of the HPR motif open the possibility that targeting of TILs to cell membranes could be mediated by interaction with other proteins. The functional analysis of the HPR motif unveils the existence of novel mechanisms involved in the intracellular targeting of proteins in plants.

**Electronic supplementary material:**

The online version of this article (doi:10.1007/s11103-015-0326-x) contains supplementary material, which is available to authorized users.

## Introduction

Lipocalins are proteins widely distributed in nature whose common feature is their ability to bind small hydrophobic molecules. Although lipocalins highly differ in their function and primary amino acid sequence all share in common the presence of an eight-stranded antiparallel β-barrel containing a hydrophobic core (Flower et al. 2000; Grzyb et al. 2006). Associated to their ability to bind hydrophobic molecules, lipocalins can fulfill a wide variety of functions such as transport of small lipophilic molecules, the regulation of immunological and developmental processes, modulation of metabolism, signal transduction and responses to stress (Akerstrom et al. 2000; Bishop 2000; Flower et al. 2000; Grzyb et al. 2006).

In contrast to the good knowledge of a number of lipocalins from bacteria, insects and mammals, much less is known about plant lipocalins. The first lipocalin-like proteins reported in plants were violaxanthin de-epoxidase and zeaxanthin epoxidase, two key enzymes of the xanthophyll cycle (Bugos et al. 1998; Hieber et al. 2000). However, the first true plant lipocalin, termed as temperature stress-induced lipocalin, was identified from wheat and Arabidopsis (Charron et al. 2002). Further data mining of EST and genomic databases revealed that plants contain two types of true lipocalins, which were classified as temperature-induced lipocalins (TILs) and chloroplastic lipocalins (CHLs) (Charron et al. 2005). Phylogenetic analysis revealed that TILs share significant homology with the bacterial outer membrane lipoprotein (Blc), the insect protein Lazarillo and the mammalian apolipoprotein D (ApoD), thus suggesting a common evolutionary origin of these proteins (Charron et al. 2005). Arabidopsis TIL (AtTIL) presents a systemic expression in several tissues and development stages (Schmid et al. 2005), eventhough the study of this protein has been related with stress responses. Thus, several studies in Arabidopsis have shown that AtTIL plays a role in basal and acquired thermotolerance (Charron et al. 2008; Chi et al. 2009), salt stress (Abo-Ogiala et al. 2014), and drought and high light stresses (Boca et al. 2014; Levesque-Tremblay et al. 2009). AtTIL has been related with protection against oxidative stress, especially in membranous environments, although its mechanism of action is currently unknown (Abo-Ogiala et al. 2014; Boca et al. 2014; Charron et al. 2008; Chi et al. 2009; Levesque-Tremblay et al. 2009).

Lipocalins Blc, ApoD and Lazarillo are known to be associated with membranes. The similarity of TILs with Blc, ApoD and Lazarillo suggested that the plant counterparts also were membrane-associated proteins (Charron and Sarhan 2005). Early studies on the subcellular localization of AtTIL indicated that this protein was mainly localized in the plasma membrane (Charron et al. 2005). However, cell fractionation studies indicate that TIL proteins may also be found in organelle enriched fractions (Abo-Ogiala et al. 2014; Boca et al. 2014; Chi et al. 2009). The intracellular translocation of TIL from the plasma membrane to the symplast in response to salt stress has recently been reported by Abo-Ogiala et al. (2014). Relocation of lipocalins in response to stress has also been reported in animal systems (Do Carmo et al. 2007). Proteomic data has shown the presence of AtTIL in tonoplast (Carter et al. 2004; Jaquinod et al. 2007), mitochondrial membranes (Brugiere et al. 2004), endoplasmic reticulum (Dunkley et al. 2006), Golgi (Nikolovski et al. 2012; Parsons et al. 2012), peroxisomes (Eubel et al. 2008) and plasmodesmata (Fernandez-Calvino et al. 2011), thereby indicating this protein may be targeted to different cell membranes and organelles. Regardless of its subcellular localization, selective extraction of proteins from membrane fractions indicated that AtTIL behaves as a peripheral membrane protein (Chi et al. 2009).

The interaction of AtTIL with cell membranes is intriguing considering that, in contrast with Blc and Lazarillo, this protein does not contain any recognizable signal for membrane targeting. Furthermore, TILs do not contain hydrophobic regions having the features of transmembrane motifs. However, and by similarity with ApoD, which has been proposed to interact with the external face of the membrane through a short hydrophobic loop (Bishop 2000; Charron et al. 2005; Grzyb et al. 2006), it has been suggested that TILs could interact with the plasma membrane by means of a short hydrophobic sequence present in a loop located between β-strands 5 and 6 (Charron and Sarhan 2005). In the present work we show that this short hydrophobic loop, which contains four conserved Pro residues, is necessary and sufficient for the targeting of TIL proteins to specific cell membranes and organelles. The targeting of TIL proteins mediated by this short hydrophobic proline-rich sequence (termed as HPR motif) uncovers a novel mechanism for the intracellular targeting of proteins in plants.

## Materials and methods

### Constructs for the expression of AtTIL, SlTIL1 and SlTIL2 fused to YFP

Temperature-induced lipocalins (TIL) constructs used in this work were generated using standard TOPO and Gateway cloning systems. The coding region of AtTIL, SlTIL1 and SltTIl2 were first amplified by RT-PCR using Pfu DNA Polymerase (Promega) and cloned into pENTR™*/*D-TOPO^®^ (Invitrogen). Primers AtTIL-Fw (CAC CAT GAC AGA GAAGAA AGA GAT GGA AG) and AtTIL-Rv1 (ATA AGT CTC GAG TCG TGT GGT GTG) were used for the amplification of the full length AtTIL coding region. The PCR product was cloned into the pENTR*/*D-TOPO to generate plasmid pENTR/D-TOPO-AtTIL, which in turn was used as a substrate of a LR reaction with the pEarlyGate104 vector (Earley et al. 2006). The generated plasmid (pYFP:AtTIL) contained the coding region of YFP upstream of the coding region of AtTIL under the control of the CaMV 35S promoter. A version of the AtTIL coding sequence without the stop codon was also generated using primers AtTIL-Fw and AtTIL-Rv2 (TTT GCC GAA GAG AGA TTT GAA CCA C). The amplification product was cloned into pENTR*/*D-TOPO as described above and then into pEarlyGate101 vector (Earley et al. 2006) to generate a construct (pAtTIL:YFP) containing the coding region of YFP fused at 3′ of the coding region of AtTIL under the control of the CaMV 35S promoter. The same strategy was used to generate the equivalent construct for SlTIl1 (pYFP:SlTIL1 and pSlTIL1:YFP) and SlTIL2 (pYFP:SlTIL2and pSlTIL1:YFP). The primers used were SlTIL1-Fw (CAC CAT GGCTAC AAA AGTAATGGAAGT G), SlTIL-Rv1 (GTC GAC GTT TTT GCC TAT TTTCCAAGG ATT G) and SlTIL1-Rv2 (GTG GAT CAA ATC AAT CCT TGG AAA A) for SlTIL1, and SlTIL2-Fw (CAC CAT GAC CAC AAA AGA GAT GGA AGT AG), SlTIL-Rv1 (CTA TTT TCC CAA TAT TGA TTT GAT CC) and SlTIL-Rv2 (GTC GAC CAT ATT CAA GAT TGA CTA TTT TCC C) for SlTIL2. The control vector expressing YFP under the control of the CaMV 35S promoter was generated by amplification of the YFP coding sequence contained in the pEarlyGate104 vector using primers YFP-Fw (CAC CAT GGG CAA GGG CGA GGA GCT GTT C) and YFP-Rv (TCA TGA TCC CGG GCC CGC GGT ACC GTC GAC). The obtained amplification product was first cloned into pENTR*/*D-TOPO and then into the pEarlyGate100 vector (Earley et al. 2006) to generate pENTR/D-TOPO-YFP.

### In vitro mutagenesis

In vitro mutagenesis was performed using overlapping PCR. The pAtTIL(SAPAR) construct was prepared using a set of 4 primers (A, B, C and D) and plasmid pAtTIL:YFP as a template. Primers A and D were M13F and M13R, provided in the pENTR™/D-TOPO^®^ Cloning Kit (Invitrogen). Primers B (GTC TCC GGT GAC GGG TCG AGC AGG CGC AGA AGG AGG GAC ATA GAA CTT G) and C (CAA GTT CTA TGT CCC TCC TTC TGC GCC TGC TCG ACC CGT CAC CGG AGA C) were designed to incorporate the SAPAR mutation. In separated PCR reactions, fragments were amplified using primers A/B and C/D. The amplification products were mixed and used as templates for a PCR reaction performed using primers A and D. The resulting amplification product was cloned into pEarlyGate101 to generate pAtTIL(SAPAR). The same strategy was used to generate the YFP derivative YFP(HPR) and the AtTIL derivatives AtTIL-P91 V:YFP, AtTIL-P92 V:YFP, AtTIL-P95 V:YFP and AtTIL-P98 V:YFP containing mutations in the proline residues present in the HPR motif. The primers (B and C) and the templates used in each case are specified in Online Resource 1.

### Protein subcellular localization in *N*. *benthamiana* leaves

For transient expression of YFP constructs, leaves of 4–6-week-old *N*. *benthamiana* plants grown at 22 °C under 16 h light/8 h dark regime were used for agroinfiltration. Plasmids were transformed into *A. tumefaciens* (C58 GV2260) cells by electroporation. The transformants were selected on agar plates containing ampicillin (100 μg/mL), kanamycin (100 μg/mL) and rifampicin (50 μg/mL). A single colony taken directly from the agar plates was grown in YEB liquid medium containing the same antibiotics. After incubation at 28 °C for 48 h the culture was centrifuged at 5000×*g* for 10 min. Cells were resuspended in 10 mM MgCl_2_, 10 mM MES pH 5.6 and 200 μM acetosyringone, adjusted at the appropriate optical density (OD) at 600 nm and incubated at room temperature for 4 h. Before agroinfiltration samples were mixed with one volume of *A. tumefaciens* (C58 GV3310) cells harbouring plasmid pICPPV-HCPVY (Goytia et al. 2006) at the same OD. The OD of the agroinfiltrated *Agrobacterium* suspensions was determined experimentally in each case to get the best fluorescence emission. For co-localization studies, cultures containing constructs YFP:AtTIL, AtTIL:YFP and YFP(HPR) were mixed with the culture containing the CFP construct of the required cell marker before infiltration. The constructs containing the cell markers fused to CFP used were PM-CK pBIN20 (plasma membrane), VAC-CK pBIN20 (tonoplast), PX-CK pBIN20 (peroxisome), MT-CK pBIN20 (mitochondria), G-CB pBIN20 (Golgi) and PT-CK pBIN20 (chloroplast) (Nelson et al. 2007). The construct containing the endoplasmic reticulum CFP marker (pCSPgECFP-KDEL) was obtained by Torrent et al. (2009). The *Agrobacterium* cell suspension was infiltrated into the abaxial side of *N. benthamiana* leaves using a 1-mL syringe. Plants were maintained in the greenhouse for 3–4 days before fluorescence observation.

### Confocal microscopy

Confocal laser scanning microscopy was performed using an Olympus FluoView FV1000 inverted confocal microscope. The 457-nm line of an argon laser was used to excite CFP and chlorophyll and the 514-nm line was used to excite YFP. The detection filters were set at 482–435 nm for CFP and 505–530 nm for YFP emission. Images were obtained and processed using software FV10-ASW (Ver4.1) (Olympus).

### Subcellular fractionation and western blot analysis

Approximately 1 g of 3–4 days *N. benthamiana* agroinfiltrated leaf zones were cut in small pieces (about 1 cm), mixed with 10 mL of ice cold lysis buffer (100 mM Hepes-Tris, 300 mM sucrose, 5 mM EDTA, 2.5 mM DTT, 1 mM PMSF and 0.5 % BSA, pH 7.4) and homogenized using an Ultra Turrax homogenizer (two pulses of 1 min on ice). The homogenized tissue was filtered through two layers of Miracloth (Calbiochem) and centrifuged in a fixed-angle rotor at 1000×*g* for 10 min at 4 °C. The supernatant (S1) was recovered and kept on ice. An aliquot of 5 mL of the S1 fraction was then centrifuged at 14,000×*g* for 10 min at 4 °C to obtain a pellet (P14) and a supernatant (S14). P14 fraction was resuspended in 5 mL of lysis buffer and kept on ice. An aliquot of 4 mL of S14 was centrifuged at 105,000×*g* for 60 min at 4 °C in a swinging bucket rotor to obtain a pellet (P105) and a supernatant (S105). The S105 fraction was withdrawn and kept on ice. The P105 pellet was washed with an equal volume of lysis buffer and centrifuged again at 105,000×*g* for 60 min at 4 °C. Finally, the P105 pellet was resuspended in lysis buffer containing 1 % SDS. 50 µL of each fraction were treated with one volume of Laemmli sample buffer and heated at 100 °C for 2 min.

For western-blot analysis, 15 µL of S1, P14, S14, P105 and S105 samples were separated by SDS-PAGE (15 % acrylamide), transferred to a nitrocellulose membrane, and probed using a rabbit anti-GFP antibody (kindly provided by Dr. D. Ludevid) at a 1:1000 dilution. Secondary anti-rabbit radish peroxidase conjugated antibody was used at a 1:5000 dilution. Chemiluminescence was detected using the Luminata Forte Western HRP Substrate (Millipore) and observed in a ImageQuant LAS4000 imager (GE Healthcare).

### In silico modeling of TIL proteins

The TIL protein models used in this work were obtained using SWISS-MODEL in automated mode (Arnold et al. 2006). For membrane-protein interactions, the predicted model was orientated using PPM web server from the Orientations of Proteins in Membranes (OPM) database (Lomize et al. 2006). Afterwards, the membrane binding simulation was performed with Membrane Automated Builder Algorithm of Database of Protein-Membrane Complexes CHARMM-GUI (Jo et al. 2007, 2008) using different lipid membrane compositions.

Accession numbers of sequence data: AtTIL, gi-15242942; SlTIL1, gi-350539735 and SlTIL2, gi-350539918.

## Results

### TIL proteins contain a conserved short hydrophobic proline-rich motif

**Fig. 1:**
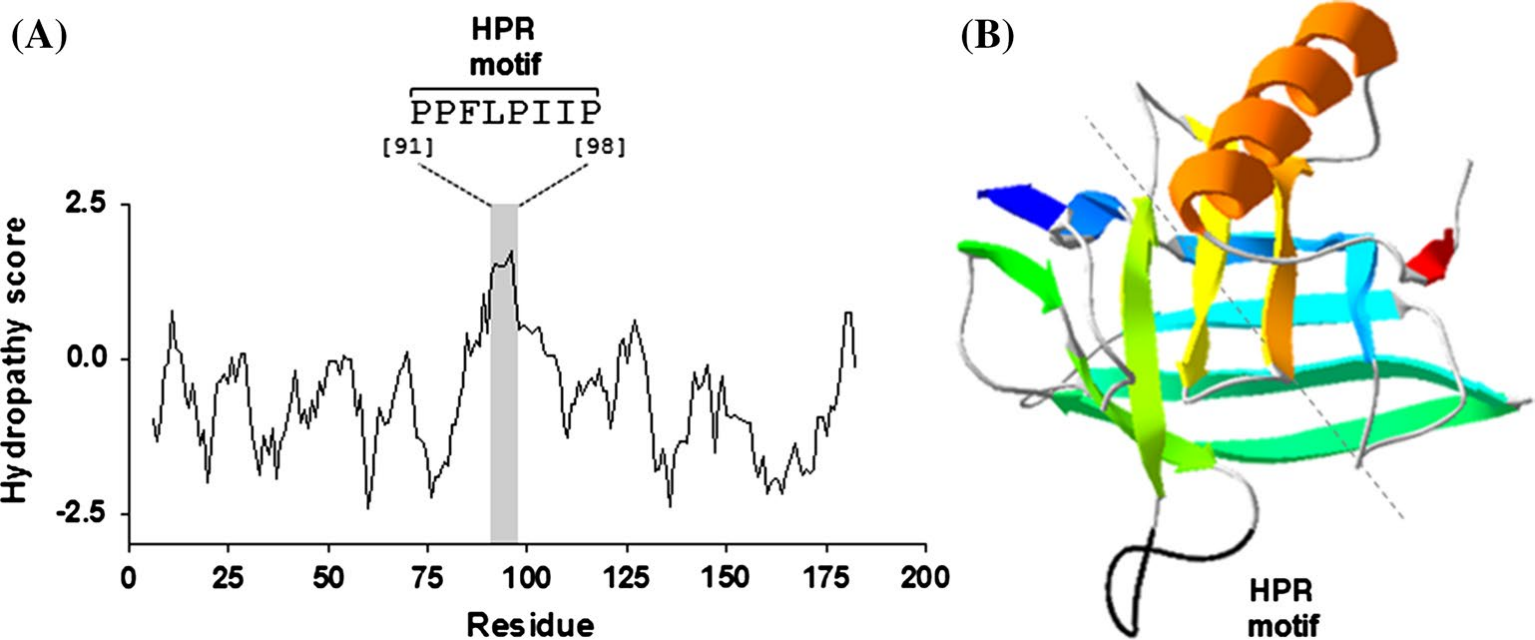
AtTIL contains a short hydrophobic proline-rich sequence (HPR motif) forming a protruding loop between β-strands 5 and 6. **a** Hydropathy plot (Kyte and Doolittle) of AtTIL showing the position and primary sequence of the HPR motif. **b** Model of the tertiary structure of AtTIL obtained using the Swiss-Model Program (Arnold et al. 2006). *E. coli* BCL protein (PDB ID:2ACO Chain A) was used as template. The HPR motif is shown in *black*. The *dashed line* indicates the axis of the lipocalin β-barrel

ESTs and genes encoding TIL proteins have been identified in different plant species (Charron et al. 2005). Whereas some plants, like Arabidopsis, contain a single gene encoding TIL, in most plants TILs are encoded by small gene families. TIL proteins show an identity of 39.4–99,1 % and their phylogenetic relationships have been reviewed by Charron et al. (2005). It has been proposed that TILs interact with cell membranes through a short hydrophobic sequence present in a loop located between β-strands 5 and 6 (Charron et al. 2005). The hydropathy profile of plant TIL proteins, exemplified by Arabidopsis TIL (AtTIL) in Fig. 1a, shows that this loop extends over a short stretch of eight hydrophobic residues (PPFLPIIP in the case of AtTIL). The alignment of TIL proteins currently available from public databases shows that this sequence is highly conserved and contains four invariant proline residues (consensus sequence ϕPPϕϕPϕϕPϕ, were ϕ represents a hydrophobic residue) (Online Resource 2). As previously described for wheat TIL (Charron et al. 2002), modeling of AtTIL and the two tomato TIL isoforms (SlTIL1 and SlTIL2) confirmed that this short hydrophobic proline-rich sequence (hereafter referred to as HPR motif) forms a loop protruding from the β-barrel (Fig. 1b and Online Resource 3).

### The HPR motif is required for the intracellular targeting of TIL proteins

**Fig. 2:**
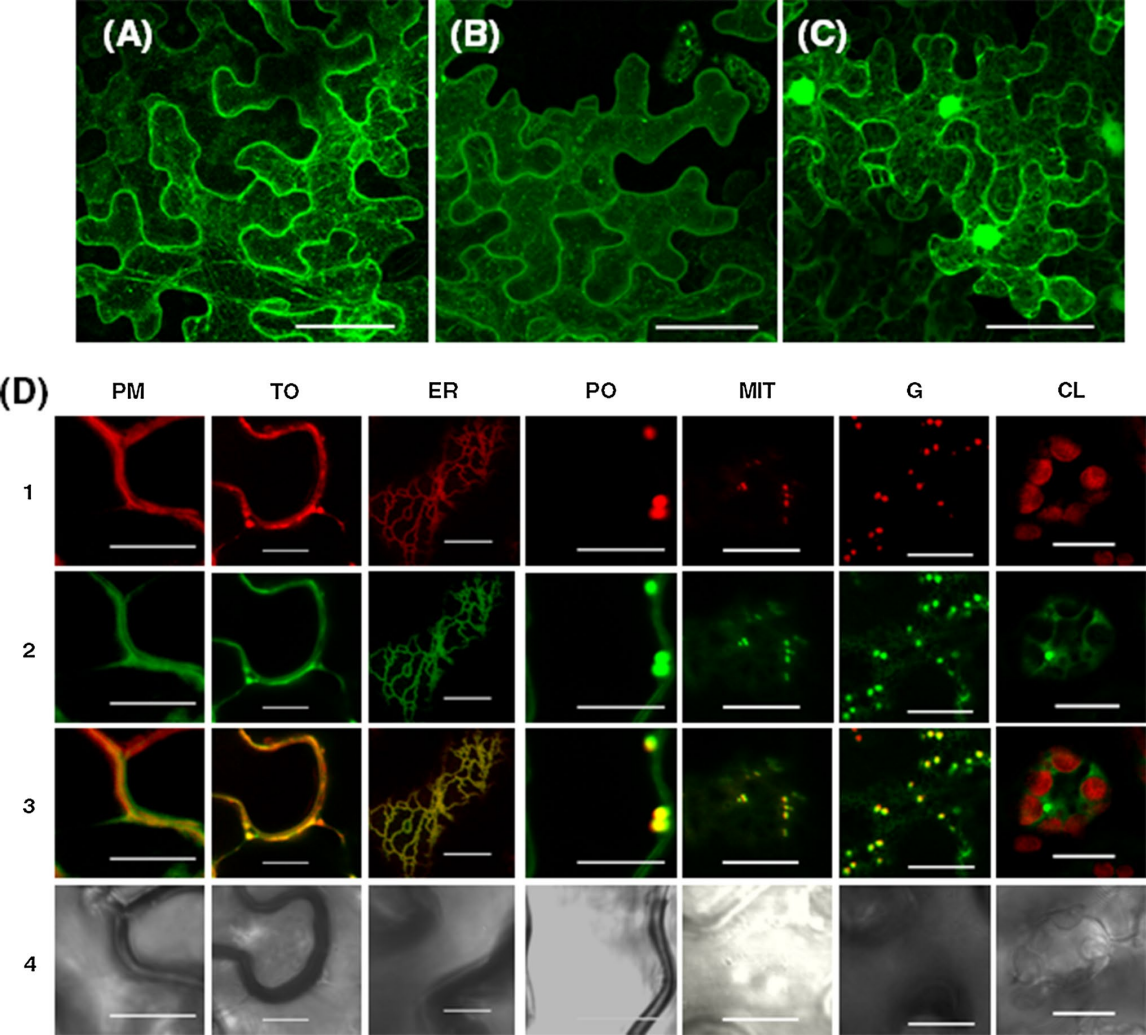
AtTIL is targeted to different cell membranes and organelles. **a**–**c** *N. benthamiana* leaves were agroinfiltrated for the transient expression of **a** AtTIL:YFP, **b** YFP:AtTIL and **c** YFP. Cells were imaged at 3 days post-infiltration. Images were reconstructed by superposition of a series of confocal optical sections. *Scale bar* 50 μm. **d** *N. benthamiana* leaves were agroinfiltrated for the co-expression of AtTIL:YFP and CFP markers for plasma membrane (PM), tonoplast (TO), endoplasmic reticulum (ER), peroxisome (PO), mitochondria (MIT), Golgi (G) and plastids (CL). Numbers on the left correspond to: *1* fluorescence of CFP, *2* fluorescence of YFP, *3* merge of images 1 and 2, and 4) bright field. Each image corresponds to a single confocal optical section. *Scale bar* 10 μm

The functional analysis of the HPR motif in the intracellular targeting of TIL proteins was performed in *Nicotiana benthamiana* leaves transiently expressing TIL proteins fused to the enhanced yellow fluorescent protein (YFP). The intracellular localization of AtTIL derivatives containing YFP fused to either the C- or the N-terminal end (AtTIL:YFP and YFP:AtTIL, respectively) is shown in Fig. 2a, b. In both cases fluorescence was detected not only in the plasma membrane (as previously described) but also in reticulate and punctate structures. Similar results were obtained when SlTIL1:YFP, YFP:SlTIL1, SlTIL2:YFP and YFP:SlTIL2 were used (Online Resource 4). In control cells expressing YFP, fluorescence was found in the cytosol and the nucleus (Fig. 2c). To better define the intracellular localization of AtTIL we performed co-localization studies using enhanced cyan fluorescent protein (CFP) derivatives containing targeting sequences to the plasma membrane, tonoplast, Golgi, peroxisomes, mitochondria and chloroplasts (Nelson et al. 2007) and the CFP-KDEL marker for endoplasmic reticulum (Torrent et al. 2009). The results shown in Fig. 2d revealed that AtTIL:YFP co-localized with plasma membrane, tonoplast, endoplasmic reticulum, Golgi, peroxisomal and mitochondrial markers. High co-localization coefficients were found in all cases as estimated using the Coloc2-Fiji program (Online resource 5) (Schindelin et al. 2012). AtTIL:YFP was not detected in chloroplasts. Equivalent results were obtained when SlTIL1:YFP was used (Online Resource 6).

**Fig. 3:**
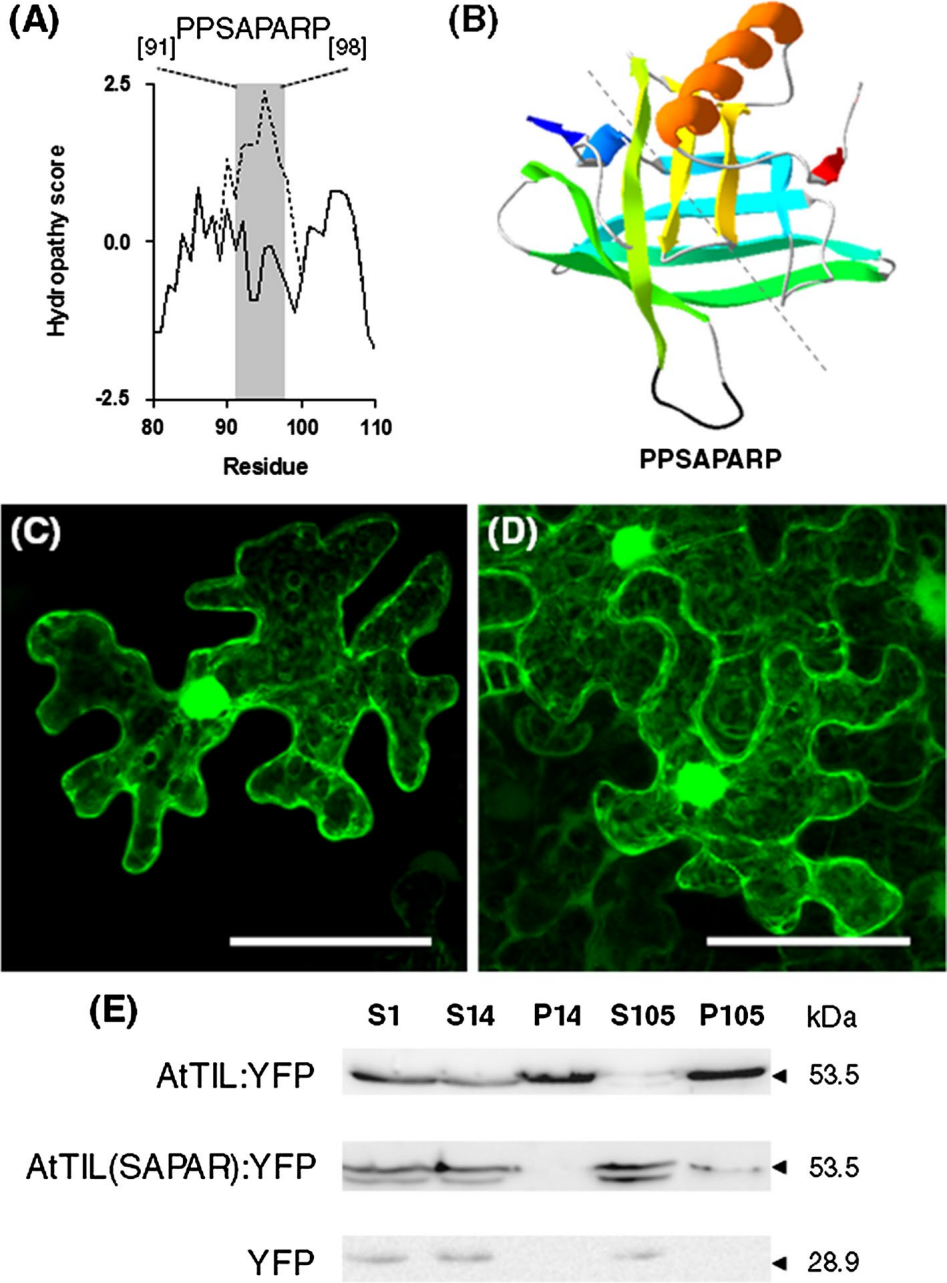
Intracellular targeting of AtTIL depends on the hydrophobic character of the HPR motif. **a** Hydropathy plot (Kyte and Doolittle) of the region extending from residues 80–110 of AtTIL(SAPAR) (*solid line*) and AtTIL (*dashed line*). The region corresponding to the HPR motif is highlighted in *grey*. **b** Model of the tertiary structure of AtTIL(SAPAR) obtained using the Swiss-Model Program (Arnold et al. 2006). *E. coli* BCL protein (PDB ID:2ACO Chain A) was used as template. The *dashed line* indicates the axis of the lipocalin β-barrel. **c**–**d** *N. benthamiana* leaves were agroinfiltrated for the transient expression of **c** AtTIL(SAPAR):YFP and **d** YFP. Cells were imaged at 3 days post infiltration. Images are reconstructed by superposition of a series of confocal optical sections. *Scale bar* 50 μm. **e** Immunoblot detection of AtTIL:YFP, AtTIL(SAPAR):YFP and YFP in cell fractions derived from agroinfiltrated *N. benthamiana* leaves. S1, 1000×*g* supernatant; S14, 14,000×*g* supernatant; P14, 14,000×*g* pellet; S105, 105,000×*g* supernatant and P105, 105,000×*g* pellet. The molecular weight of the identified proteins is shown on the *right*

To evaluate the possible contribution of the HPR motif in the intracellular targeting of TIL proteins we first performed transient expression assays using an AtTIL derivative in which the HPR motif was mutated to eliminate its hydrophobic character. Considering the potential structural role of the conserved proline residues, the changes were made to the other hydrophobic residues. The mutant version of the HPR motif PPSAPARP (amino acid changes are underlined) can form a loop with a similar folding but lacking the strong hydrophobic character of the HPR motif (Fig. 3a, b). When the mutant version of AtTIL containing the PPSAPARP sequence, hereafter referred to as AtTIL(SAPAR) was fused to YFP and transiently expressed in *N. benthamiana* leaves the distribution pattern was similar to that of YFP. In both cases fluorescence was visualized in the cytosol and the nucleus (Fig. 3c, d). The differential localization of AtTIL(SAPAR):YFP with respect to AtTIL:YFP was confirmed by immunoblot analysis (Fig. 3e). These results indicate that the hydrophobic character of the HPR motif plays a major role in the intracellular targeting of AtTIL.

### The HPR motif targets YFP to cells membranes

**Fig. 4:**
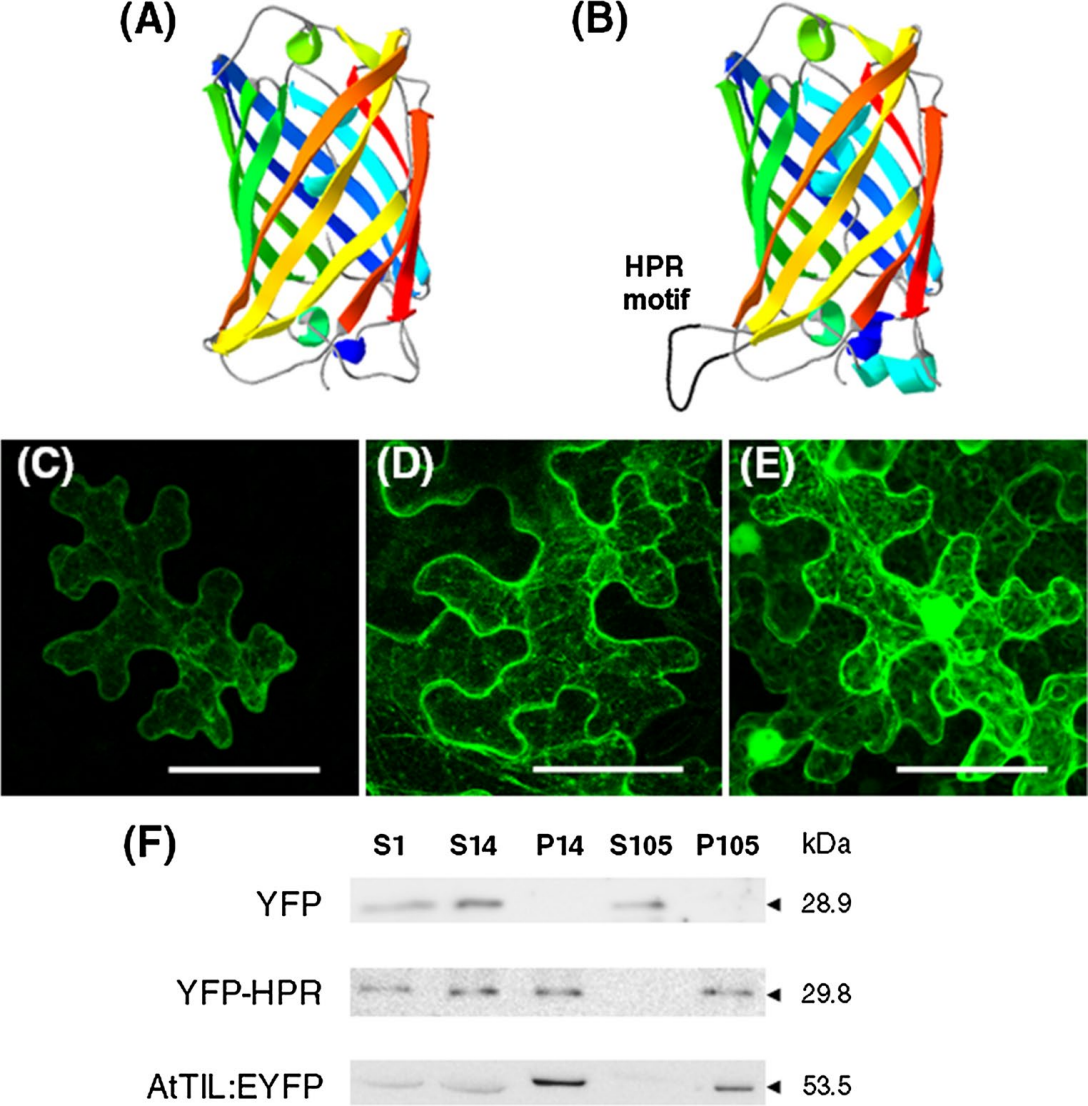
The HPR motif is sufficient for the targeting of YFP to cell membranes and organelles. **a** Tertiary structure of GFP (Royant and Noirclerc-Savoye 2011). **b** Tertiary structure model of YFP-HPR obtained from Swiss-Model Program using GFP (PDB ID:2Y0G) as a template. **c**–**e** *N. benthamiana* leaves were agroinfiltrated for the transient expression of **c** YFP-HPR, **d** AtTIL:YFP and **e** YFP. Cells were imaged at 3 days post infiltration. Images are reconstructed by superposition of series of confocal optical sections. *Scale bar* 50 μm. **f** Immunoblot detection of YFP, YFP-HPR and AtTIL:EYFP in cell fractions derived from agroinfiltrated *N. benthamiana* leaves. S1, 1000×*g* supernatant; S14, 14,000×*g* supernatant; P14, 14,000×*g* pellet; S105, 105,000×*g* supernatant and P105, 105,000×*g* pellet. The molecular weight of the identified proteins is shown on the right

To get further insights into the role of the HPR motif in the intracellular targeting of TIL proteins, a strategy based on the use of YFP as scaffold for the intracellular presentation of peptide motifs was used (Abedi et al. 1998). To this end, we generated a variant of YFP in which the amino acid residue Q157, present in the exposed loop between β-strands 6 and 7, was replaced by the sequence VPPFLPIIPV containing the HPR motif (underlined). Modeling of the modified YFP protein (YFP-HPR) shows that the HPR motif appears as a protruding loop (Fig. 4b) similar to that predicted for AtTIL, SlTIL1 and SlTIL2.

When YFP-HPR was transiently expressed in *N. benthamiana* leaves the fluorescence pattern observed was similar to that described above for AtTIL:YFP (Fig. 4c; Online Resource 7). The association of YFP-HPR with cell membranes and organelles was confirmed by immunoblot analysis (Fig. 4f). These results indicate that the HPR motif is sufficient for targeting proteins to cell membranes.

### Role of the proline residues present in the SPHR motif

**Fig. 5:**
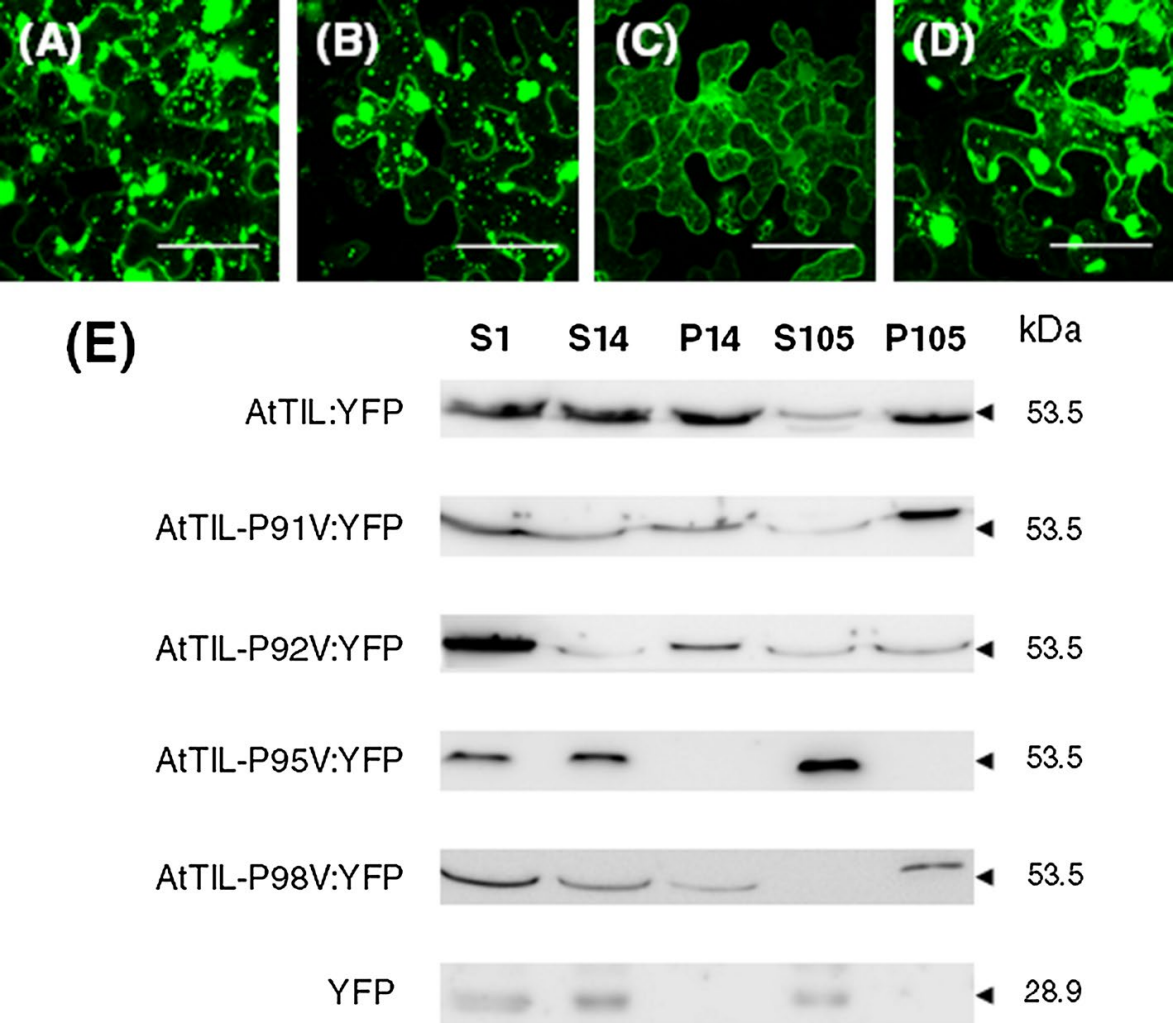
Role of the proline residues present in the HPR motif in the intracellular targeting of AtTIL. **a**–**d** *N. benthamiana* leaves were agroinfiltrated for the transient expression of AtTIL-**a** P91V:YFP, **b** AtTIL-P92V:YFP, **c** AtTIL-P95 V:YFP and **d** AtTIL-P98 V:YFP. Cells were imaged at 3 days post infiltration. Images are reconstructed by superposition of a series of confocal optical sections. *Scale bar* 50 μm. **e** Immunoblot detection of AtTIL:YFP, AtTIL-P91V:YFP, AtTIL-P92V:YFP, AtTIL-P95V:YFP and AtTIL-P98V:YFP and YFP in cell fractions derived from agroinfiltrated *N. benthamiana* leaves. S1, 1000×*g* supernatant; S14, 14,000×*g* supernatant; P14, 14,000×*g* pellet; S105, 105,000×*g* supernatant and P105, 105,000×*g* pellet. The molecular weight of the identified proteins is shown on the right

The alignment the TIL proteins has shown that the HPR motif contains four conserved proline residues (Online Resource 2). This feature suggested that these amino acids might have a relevant role in the structure or function of the HPR motif. To investigate the role of these proline residues (P91, P92, P95 and P98) we generated AtTIL:YFP derivatives in which each one of the proline residues present in the HPR motif was replaced by valine (AtTIL-P91 V:YFP, AtTIL-P92 V:YFP, AtTIL-P95 V:YFP and AtTIL-P98 V:YFP). Transient expression of these proteins in *N. benthamiana* leaves revealed that the P95 mutation had a major effect on the intracellular targeting of AtTIL, which showed a localization pattern resembling that of YFP (Fig. 5c). Immunoblot analysis confirmed the absence of AtTIL-P95 V:YFP in the organellar (P14) and microsomal (P105) fractions (Fig. 5e). In the case of the variants harboring mutations P91 V, P92 V or P98 V, the fluorescence pattern was similar to that of AtTIL:YFP (Fig. 5 a, b, d). However, it is worth noting the presence of a large number of vesicles or membranous aggregates of unknown nature in the cells expressing AtTIL-P91 V:YFP, AtTIL-P92 V:YFP and AtTIL-P98 V:YFP (Fig. 5a, b, d). These results suggest that P91, P92 and P98 may also have a role in the right targeting of TIL proteins to cell membranes.

### The HPR motif is also present in other Arabidopsis proteins

To elucidate whether the HPR motif is unique to TIL proteins, a databank search was performed to identify the eventual presence of equivalent sequences in unrelated plant proteins. A Pattern Matching Tool (www.arabidopsis.org) search performed against the Arabidopsis genome database retrieved 11 gene loci encoding proteins containing the consensus sequence of the HPR motif (PPϕϕPϕϕP). In addition to AtTIL, the list includes six members of the UDPG-glucosyltransferase family (At1g07250, At1g07260, At1g22380, At2g29740, At3g21790, and At4g01070), one member of the hydroxyproline-rich glycoprotein family protein (At1g14710), one splicing factor-PWI domain-containing protein (At1g60200), one member of the galactose oxidase/kelch repeat superfamily protein (At1g55270) and a protein of unknown function (At5g64540). Secondary structure prediction of these proteins indicates that in all cases the PPϕϕPϕϕP sequence may form a loop. With the only exception of the UDPG-glucosyltransferase UGTt2B1 (encoded by the At4g01070 gene), which has been characterized at functional level (Brazier-Hicks and Edwards 2005), nothing is known about the biological function or the subcellular localization of the rest of the proteins.

## Discussion

Our studies on the subcellular localization of AtTIL fused to YFP in agroinfiltrated *N. benthamiana* leaves have shown that this protein is targeted to different cell membranes and organelles. In particular, we have found the association of AtTIL with the plasma membrane, endoplasmic reticulum, tonoplast, Golgi vesicles, mitochondria and peroxisomes (Fig. 2). These results were unexpected considering previous reports showing that equivalent TIL fusion proteins mainly localized in the plasma membrane when using transfected onion epidermal cells and Arabidopsis protoplasts (Abo-Ogiala et al. 2014; Charron et al. 2005). Although the detection of fluorescence in different cell membranes and organelles may be due to the over-expression of the chimeric AtTIL proteins, our results are in agreement with proteomic data reporting the presence of this protein in mitochondrial membranes (Brugiere et al. 2004), tonoplast (Carter et al. 2004; Jaquinod et al. 2007), peroxisomes (Eubel et al. 2008), endoplasmic reticulum (Dunkley et al. 2006) and Golgi (Nikolovski et al. 2012; Parsons et al. 2012). Our results have also revealed that AtTIL is not targeted to chloroplasts (Fig. 2). These results are also in accordance with the fact that AtTIL has never been reported as a component of the chloroplast proteome (Bruley et al. 2012; Ferro et al. 2010). The observation that the tomato SlTIL1 isoform shows the same subcellular localization than AtTIL (Online Resources 4 and 6) suggests that all plant TIL proteins have the same intracellular localization.

It has been proposed that TIL proteins play an important role in preventing lipid peroxidation during stress (Chi et al. 2009). However, the nature of the target membrane lipids and the mechanism of action of TILs are currently unknown. The observation that TILs do not associate with chloroplasts probably reflects that lipid composition may be a key aspect in the intracellular targeting of TIL proteins to cell membranes. In this respect, it is relevant to point out that the lipid composition of chloroplast membranes differs from the rest of cell membranes. Chloroplast membranes are unique as they contain glycolipids and have a relatively low amount of phospholipids (Block et al. 2007; Jouhet et al. 2007). Furthermore, phosphatidylethanolamine and phosphatidylserine (two common phospholipids present in most intracellular membranes) are absent in plastid membranes (Block et al. 2007). The subcellular distribution of TILs reported here indicates that TIL proteins may have a role in protecting not only plasma membrane lipids during stress, as initially proposed, but also in protecting other cell membranes having a similar lipid composition. Concerning chloroplasts, it is likely that chloroplastic lipocalin CHL may perform a similar role in the protection of plastidial membranes during stress by interacting with chloroplast lipids. This hypothesis is supported by the recent observation that Arabidopsis AtTIL and AtCHL play distinct but overlapping roles in lipid protection during stress (Boca et al. 2014).

The signals and mechanism underlying the targeting of TIL proteins to cell membranes has not been studied in detail. Furthermore, the interaction of TILs with cell membranes in an intriguing issue considering the marked hydrophilic character of these proteins (Fig. 1b; Online Resource 4a, c). However, it has been long been proposed that the short hydrophobic sequence located between β-strands 5 and 6 could of TIL proteins play a major role in their interaction with cell membranes by means (Charron et al. 2002, 2005). The structural modeling of AtTIL, SlTIl1 and SlTIL2 predicts that this sequence forms a loop that protrudes from the β-barrel (Fig. 1b and Online Resource 4a, c). This loop (referred to as HPR motif) is highly conserved among TIL proteins and contains four invariant proline residues (Online Resource 2).

By using chimeric TIL:YFP versions in which the HPR motif was mutated to introduce hydrophilic residues it was found that the hydrophobic character of the HPR motif is a key determinant for the intracellular targeting of TIL proteins (Fig. 3). We have also shown that a version of YFP containing the HPR motif has a subcellular localization similar to that of TIL-YFP proteins (Fig. 4). Taken together, these results indicate that the HPR motif is both necessary and sufficient for the intracellular targeting of TIL proteins.

As indicated above, the HPR motif contains four invariant proline residues (P91, P92, P95 and P98) (Online Resource 2). Since proline residues confer unique structural constrains on peptide chains it is likely that these conserved residues could play a relevant role in the structure or function of the HPR motif. To evaluate the role of these residues we generated AtTIL-YFP derivatives in which each one of the four proline residues was systematically replaced by valine. Transient expression of these mutant AtTIL-YFP versions showed that P95 has a major role in the targeting of AtTIL. As shown in Fig. 5, AtTIL-P95 V:YFP behaves as a cytosolic protein. Although mutations in P91, P92 and P98 had no significant effects on the intracellular targeting of the corresponding AtTIL derivatives, the expression of these AtTIL variants induced the formation of vesicular structures or aggregates of unknown nature (Fig. 5). Taken together, these results suggest a relevant role of the proline residues (in particular P95) in the structure and/or the function of the HPR motif.

**Fig. 6:**
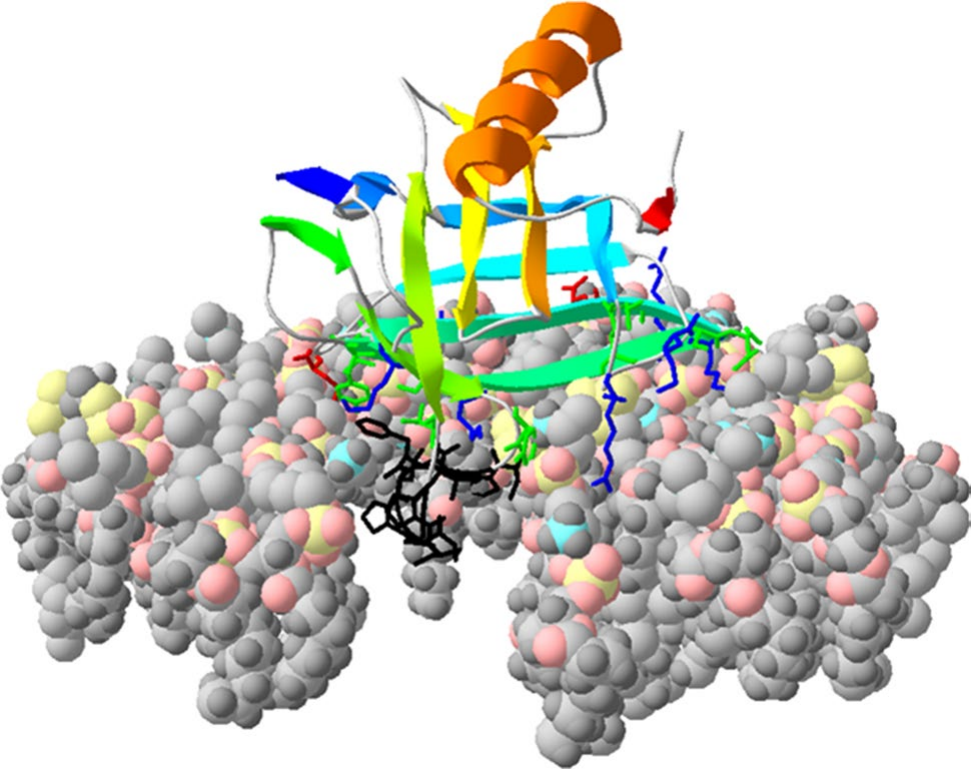
Hypothetical model of interaction between AtTIL and cell membranes. The spatial position of AtTIL inserted in model membranes was calculated in the PPM web server of the OPM database (Lomize et al. 2012). The calculated surface was used in Membrane Automated Builder Algorithm of Database Protein-Membrane Complexes CHARMM-GUI (Jo et al. 2007, 2008) to generate a membrane interacting with AtTIL. The lipid ratio of the membrane was calculated from Membrane Protein Lipid Composition Atlas of OPM database (van Meer et al. 2008). The lipid ratio composition (max–min ratio) was 22–6 % ergosterol, 48–28 % phosphatidylcholine, 31–18 % phosphatidylethanolamine, 14–5 % phosphatidyl inositol, 20–1 % phosphatidylserine, 6–2 % phosphatidic acid, 6–0 % cardiolipin, and 18–3 % sphingolipids. For simplicity only one membrane layer is shown and some lipids have been removed after modeling to allow the visualization of the HPR motif inside the membrane. Atoms in membrane lipids are shown in the following *colors*: carbon in *light grey*, oxygen in *pink*, phosphorous in *yellow*, nitrogen in *blue* and hydrogen in *dark grey*. The membrane embedded hydrophobic residues of the HPR motif are shown in *black*. Residues potentially interacting with the polar head of membrane lipids are also shown: Acidic amino acids are in red, basic residues in *blue* and polar residues in *green*

Modeling studies have shown that the HPR motif could mediate the anchoring of TIL proteins to cell membranes (Fig. 6). According to this model, the HPR motif (estimated length of about 10 Å) would be embedded into one of the membrane layers. Assuming that the function of TIL proteins during stress is to prevent the damage of membrane lipids, or eventually participate in their repair, the anchoring to cell membranes may represent the first step in their mechanism of action. Thus, it could be hypothesized that the primary anchoring of TIL proteins to cell membranes mediated by the HPR motif can facilitate their further interaction with membrane lipids. In this respect, the proposed model predicts the positioning of TIL proteins in a way that the internal cavity the β-barrel locates at the vicinity of the polar headgroups of membrane lipids, allowing in this way the eventual interaction with the target lipids. This model also predicts several polar and charged amino acids residues close to the HPR motif that could help stabilizing the interaction between TIL proteins and the polar surface of cell membranes (Fig. 6). The association of proteins to cell membranes through the specific recognition of particular membrane lipids is a well documented process (Lemmon 2008; Moravcevic et al. 2012).

Although the direct interaction of the HPR motif with cell membranes could represent the primary step in the mechanism of action of TIL proteins, the involvement of interacting proteins in this process cannot be excluded. As indicated above, one of the main features of the HPR motif is the presence of four conserved proline residues. In this regard, it is known that proline residues play a critical role in many ligands involved in protein–protein interactions (Kay et al. 2000). For instance, the sequence XPxXP (where X is usually a hydrophobic residue and x is any other residue) is present in the canonical consensus target for the binding to the Src homology 3 (SH3) domain, which represents one of the most widespread protein recognition modules (Saksela and Permi 2012). Interestingly, two such consensus motives are present in a tandem array within the HPR motif (PPFLP and LPIIP in the case of AtTIL, SlTIL1 and SlTIL2). Moreover, the only mutation in the HPR motif severely affecting the targeting of TIL proteins involves the third proline residue (P95). This mutation results in the simultaneous elimination of the two xPxXP sequences present in the HPR motif. Taking together, these observations open the possibility that TIL proteins could be alternatively targeted to cell membranes in a process mediated by the interaction with other proteins. However, and in contrast with other organisms, plants contain a reduced number of proteins containing the SH3 domain (Lam and Blumwald 2002). The Arabidopsis genome contains only eight genes encoding proteins containing the canonical SH3 domain and, at present, only three of them have been characterized. These proteins participate in vesicle trafficking and autophagy (Lam and Blumwald 2002; Zhuang et al. 2013). Other known protein interacting domains involving proline rich motifs are WW, EVH1, GYF, UEV and CAP-Gly (Adzhubei et al. 2013). However, none of them are present in the HPR motif. Therefore, further studies are needed to clarify if the targeting of TIL proteins to cell membranes may eventually involve the interaction with other proteins.

To the best of our knowledge the characterization of the HPR motif present in TIL proteins uncovers a novel mechanism for the intracellular targeting of proteins to cell membranes and organelles. However, further research is required to clarify whether the HPR motif directly anchors to cell membranes or if, alternatively, it interacts with particular membrane associated proteins.

## Electronic supplementary material

Supplementary material 1 (PDF 871 kb)
