# Supplementary material for: A hydrophobic proline-rich motif is involved in the intracellular targeting of temperature-induced lipocalin
Source: Plant Mol Biol. 2015 May 10;88(3):301–11. doi: 10.1007/s11103-015-0326-x (PMC4441748; doi:10.1007/s11103-015-0326-x)
Supplement: Supplementary file 1 — Supplementary material 1 (PDF 871 kb) [file 11103_2015_326_MOESM1_ESM.pdf]

Online Resource 1. Primers used to generate constructs AtTIL-P91V, AtTIL-P92V, AtTIL-P95V and AtTIL-P98V and YFP(HPR) using overlapping PCR. pENTR/D-TOPO-AtTIL was used as template to generate the constructs AtTIL-P91V, AtTIL-P92V, AtTIL-P95V and AtTIL-P98V. pENTR/D-TOPO-YFP was used as template to generate YFP-HPR construct.

Construct	Primer
AtTIL-P91V	AtTIL(P91V)B: GGA GGA AAG GAA CGA CAT AGA AC
	AtTIL(P91V)C: GTT CTA TGT CGT TCC TTT CCT CC
AtTIL-P92V	AtTIL(P92V)B: GAT TGG GAG GAA AAC AGG GAC ATA G
	AtTIL(P92V)C: CTA TGT CCC TGT TTT CCT CCC AAT C
AtTIL-P95V	AtTIL(P95V)B: CGG GAA TGA TTA CGA GGA AAG GAG
	AtTIL(P95V)C: CTC CTT TCC TCG TAA TCA TTC CCG
AtTIL-P98V	AtTIL(P98V)B: GTC TCC GGT GAC GAC AAT GAT TGG G
	AtTIL(P98V)C: CCC AAT CAT TGT CGT CAC CGG AGA C
YFP-HPR	YFP(HPR)B: CAG GAA TTA TAG GCA AAA ATG GTG GAA CCT TGT CGG CCA TGA TAT AGA CG
	YFP(HPR)C: TCC ACC ATT TTT GCC TAT AAT TCC TGT TAA GAA CGG CAT CAA GGT GAA C

Online Resource 2. Sequence alignment of the HPR motif of plant TIL proteins. TILs were identified using the AtTIL protein sequence as a query using BLASTP against the GenBank non-redundant protein sequence database. Only full-length sequences have been included. Amino acid residues common to all sequences are shown in black boxes. In the consensus sequence, Φ corresponds to hydrophobic residues and X to any amino acid residue.

Name	Species	Accession No.	HPR motif sequence
AtTIL	*Arabidopsis thaliana*	gi-15242942	YVPPFLPIIPVT
BdTIL1	*Bracunkodium distachyon*	gi-357147915	YVPPFLPVFPVV
BdTIL2	*Bracunkodium distachyon*	gi-357150111	YVPPILPIIPVV
BnTIL	*Brassica napus*	gi-77744889	YVPPFLPIIPVT
BrTIL	*Brassica rapa*	gi-157849740	YVPPFLPIIPVT
CaTIL	*Capsicum annuum*	gi-50236424	YVPPFLPVIPVV
CsTIL	*Citrus sinensis*	gi-77744899	YVPPFFPIIPVV
EgTIL	*Elaeis guineensis*	gi-192911934	YVPPFFPIIPVT
GaTIL	*Gossypium arboreum*	gi-77744897	YVPPFLPIIPIV
GaTIL2	*Gossypium arboreum*	gi-77744869	YVPPFLPIIPVT
GmTIL1	*Glycine max*	gi-351734470	WVPPFLPIIPVT
GmTIL2	*Glycine max*	gi-351724275	YIPPFLPIIPIN
GmTIL3	*Glycine max*	gi-351726387	WVPPFLPLFPVT
GmTIL4	*Glycine max*	gi-351721591	YVPPFLPIIPVT
HvTIL1	*Hordeum vulgare*	gi-77744845	YVPPFLPIIPVV
HvTIL2	*Hordeum vulgare*	gi-326527227	YVPPFLPVFPVT
HvTIL3	*Hordeum vulgare*	gi-77744853	YVPPFLPVFPVT
LjTIL1	*Lotus japonicus*	gi-388518293	YVPPFLPIIPVV
LjTIL2	*Lotus japonicus*	gi-388522945	FVPPFLPIIPVV
McTIL1	*Mesembryanthemum crystallinum*	gi-77744895	YVPPFLPIIPVT
McTIL2	*Mesembryanthemum crystallinum*	gi-77744867	YVPPFLPIIPVT
MtTIL1	*Medicago truncatula*	gi-357480181	LVPPFLPFIPAV
MtTIL2	*Medicago truncatula*	gi-357480173	YVPPFLPIIPAV
MtTIL3	*Medicago truncatula*	gi-357480171	YVPPMLPIIPVT
OsTIL1	*Oryza sativa japonica*	gi-115447273	YLPPFLPVIPVV
OsTIL2	*Oryza sativa japonica*	gi-115476610	YVPPFLPIFPVV
OsTIL3	*Oryza sativa japonica*	gi-151935409	YLPPFLPVIPVV
PaTIL	*Prunuspersica*	gi-77744891	YVPPFLPIIPVV
PbTIL	*Populus balsamifera*	gi-77744901	YVPPFLPIIPVV
PeTIL	*Populus euphratica*	gi-209967467	YVPPFLPIIPVV
PpTIL	*Physcomitrella patens*	gi-168066921	LVPPFFPIFPVT
PsTIL	*Piceasitchensis*	gi-116783015	MVPPFLPIIPVY
PtreTIL	*Populus tremuloides*	gi-77744903	YVPPFLPIIPVV
PtriTIL	*Populus trichocarpa*	gi-224143988	YVPPFLPIIPVV
PtTIL	*Pinus taeda*	gi-77744881	MVPPFFPIIPVY
RcTIL	*Ricinus communis*	gi-255565025	YVPPFLPIIPVV
SbTIL1	*Sorghum bicolor*	gi-242065756	YLPPFLPVIPVV
SbTIL2	*Sorghum bicolor*	gi-77744857	YVPPFLPVFPVT
SbTIL3	*Sorghum bicolor*	gi-242079289	YVPPFLPVFPVT
SlTIL1	*Solanum lycopersicum*	gi-350539735	YVPPFLPIIPVT
SlTIL2	*Solanum lycopersicum*	gi-350539918	YIPPFLPIIPIV
SmTIL1	*Selaginella moellendorffii*	gi-302802027	WVPPFLPVFPVT
SmTIL2	*Selaginella moellendorffii*	gi-302818492	WVPPFLPVFPVT
SoTIL	*Saccharum officinarum*	gi-77744875	YLPPFLPIIPVV
StTIL1	*Solanum tuberosum*	gi-77744887	YVPPFLPIIPVT
StTIL2	*Solanum tuberosum*	gi-77744865	YVPPFLPIIPVT
SuTIL	*Syntrichia ruralis*	gi-77744919	MVPPFLPVIPVT
TaTIL1	*Triticuma estivum*	gi-18650668	YVPPFLPIIPVV
TaTIL2	*Triticuma estivum*	gi-77744851	YVPPFLPVFPVT
VvTIL	*Vitis vinifera*	gi-77744883	YVPPFLPIIPVV
ZmTIL1	*Zea mays*	gi-226530914	YLPPFLPIVPVV
ZmTIL2	*Zea mays*	gi-226529802	YVPPFLPLIPVT
		**Consensus**	XΦPPΦΦPΦΦPΦX

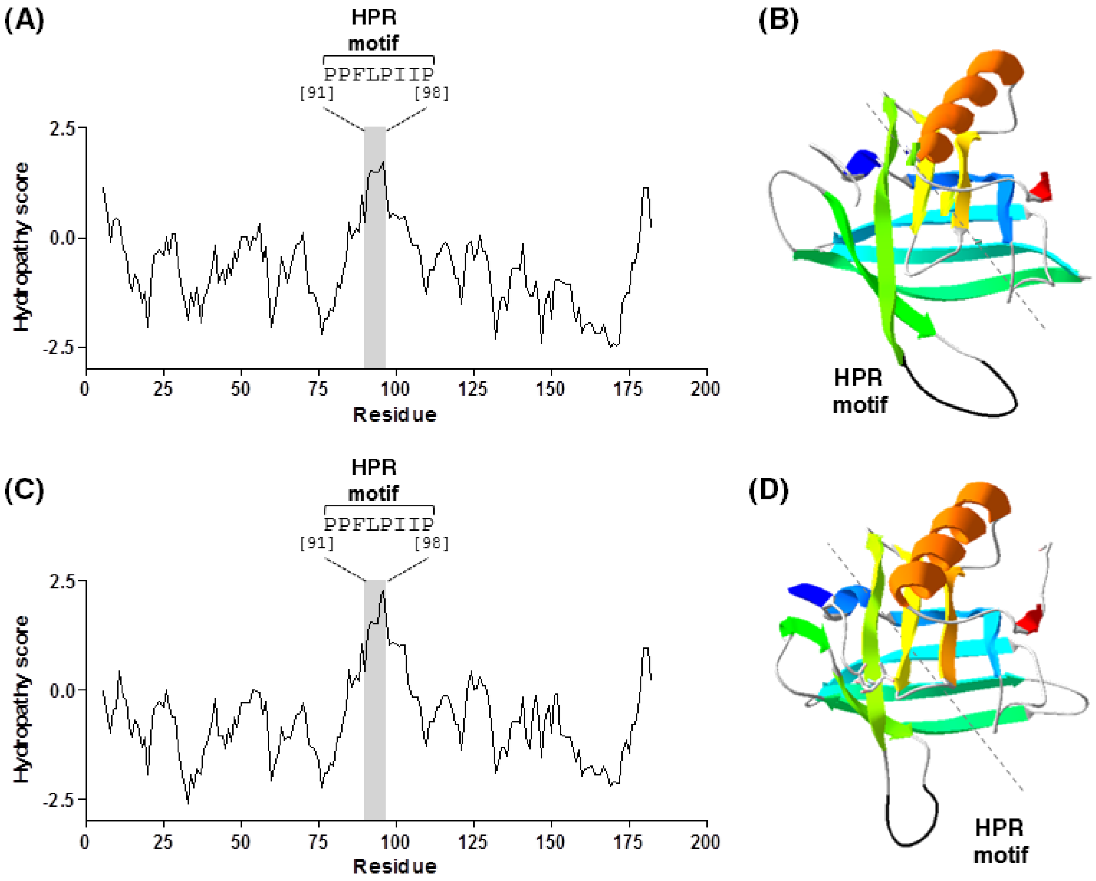

Online Resource 3. Hydropathy plot and model of the tertiary structure of SlTIL1 and SlTIL2.

a Hydropathy plot (Kyte and Doolittle) of SlTIL1 showing the position and primary sequence of the HPR motif.

b Model of the tertiary structure of SlTIL1 obtained using the Swiss-Model Program (Arnold *et al.,* 2006). *E. coli* BCL protein (PDB ID:2ACO Chain A) was used as template. The dashed line indicates the axis of the lipocalin β-barrel.

c Hydropathy plot (Kyte and Doolittle) of SlTIL2 showing the position and primary sequence of the HPR motif.

d Model of the tertiary structure of SlTIL2 obtained using the Swiss-Model Program (Arnold *et al.,* 2006). *E. coli* BCL protein (PDB ID:2ACO Chain A) was used as template. The dashed line indicates the axis of the lipocalin β-barrel.

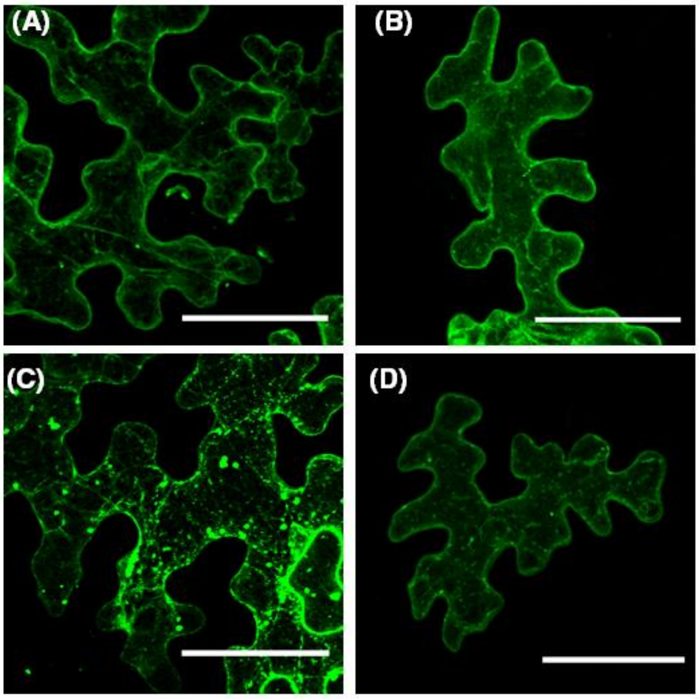

Online Resource 4. Subcellular localization of SlTIL1 and SlTIL2 in agroinfiltrated *N benthamiana* leaves.

N. benthamiana leaves were agroinfiltrated for the transient expression of: **a** YFP:SlTIL1, **b** SlTIL1:YFP, **c** YFP:SlTIL2 and **d** SlTIL2:YFP. Cells were imaged at 3 days post infiltration. Images are reconstructed by superposition of series of confocal optical sections. Scale bar = 50 µm.

Online Resource 5. Co-localizacion coefficients of the fluoresence of AtTIL-YFP and the corresponding subcellular markes shown in Figure 2D.

Co-localization coefficients were analyzed as described in program Coloc2-Fiji (http://fiji.sc/Coloc_2) (Schindelin et al. 2012). PM, plasma membrane; TO, tonoplast; ER, endoplasmic reticulum; PO, peroxisome; MIT, mitochondria; G, Golgi and CL, plastids.

	PM	TO	RE	PO	MIT	G	CL
Pearson's r coefficient	0.81	0.71	0.8	0.84	0.84	0.76	-0.31
Manders coefficient M1	0.999	1	0.879	1	0.906	1	0
Manders coefficient M2	0.885	0.996	0.982	0.59	0.443	0.406	0.513

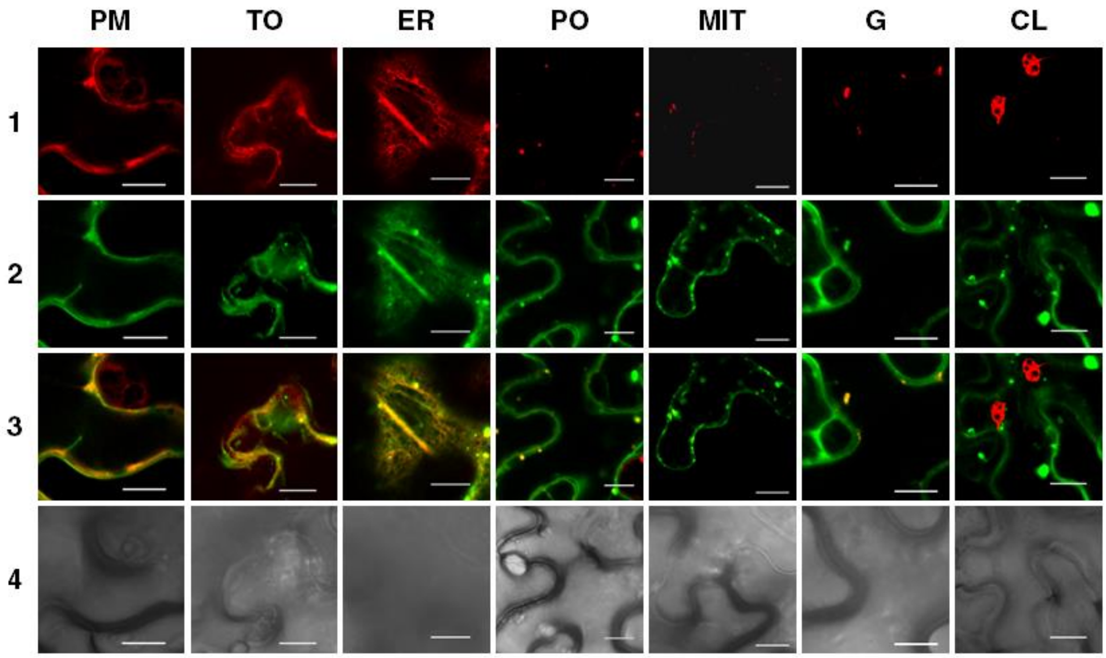

Online Resource 6. Co-localization of SlTIL1:YFP with cell markers in agroinfiltrated *N benthamiana* leaves.

N. benthamiana leaves were agroinfiltrated for the co-expression of SlTIL:YFP and markers for plasma membrane (PM), tonoplast (TO), endoplasmic reticulum (ER), peroxisome (PO), mitochondria (MIT), Golgi (G) and plastids (Cl). Numbers on the left-hand side correspond to: **1** fluorescence of CFP intracellular markers, **2** fluorescence of SlTIL:YFP, **3** merge of images from **1** and **2**, and **4** bright field. Each image corresponds to a single confocal optical section. Scale bar = 10 μm.

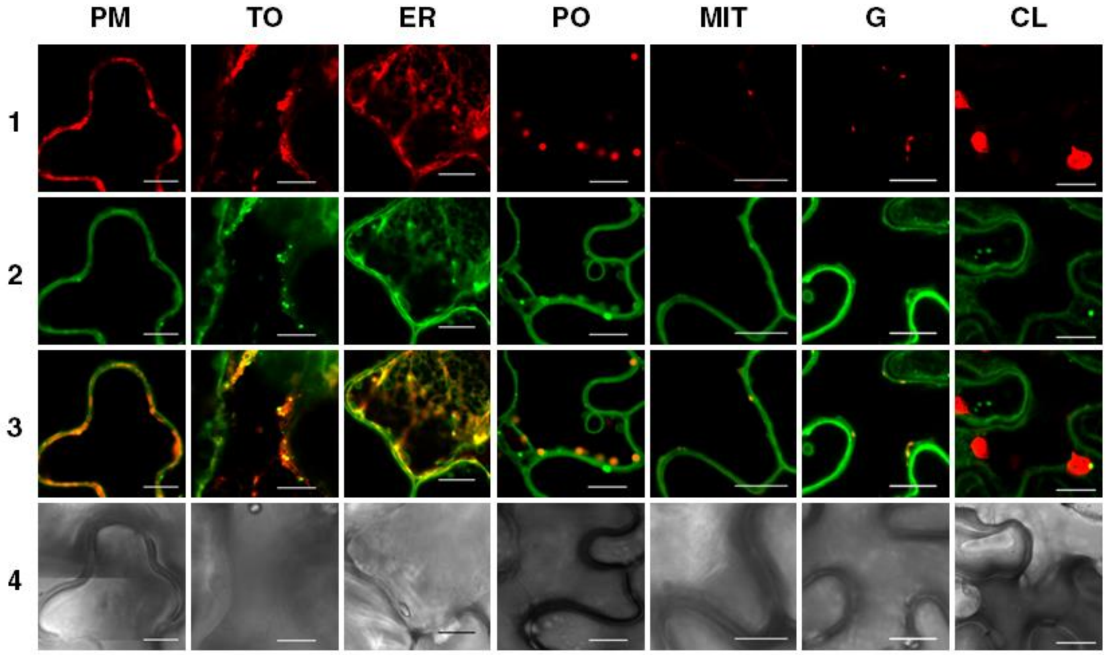

Online Resource 7. Co-expression of YFP(HPR) with cell markers in agroinfiltrated N benthamiana leaves.

N. benthamiana leaves were agroinfiltrated for the co-expression of YFP-HPR with of SlTIL:YFP and markers for plasma membrane (PM), tonoplast (TO), endoplasmic reticulum (ER), peroxisome (PO), mitochondria (MIT), Golgi (G) and plastids (Cl). Numbers on the left-hand side correspond to: **1** fluorescence of CFP intracellular markers, **2** fluorescence of YFP-HPR, **3** merge of images from **1** and **2**, and **4** bright field. Each image corresponds to a single confocal optical section. Scale bar = 10 µm.
